# Toward nutrition improving outcome of critically ill patients: How to interpret recent feeding RCTs?

**DOI:** 10.1186/s13054-023-04317-9

**Published:** 2023-01-27

**Authors:** Jan Gunst, Michael P. Casaer, Jean-Charles Preiser, Jean Reignier, Greet Van den Berghe

**Affiliations:** 1 Clinical Division and Laboratory of Intensive Care Medicine, Department of Cellular and Molecular Medicine, KU Leuven, Herestraat 49, 3000 Leuven, Belgium; 2 Erasme University Hospital, Université Libre de Bruxelles, Brussels, Belgium; 3 Service de Médecine Intensive Réanimation, Centre Hospitalier Universitaire de Nantes, Université de Nantes, Nantes, France

**Keywords:** Critical illness, Enteral nutrition, Parenteral nutrition, Amino acid, Indirect calorimetry, Energy target, Autophagy, Ketone, Intermittent feeding

## Abstract

Although numerous observational studies associated underfeeding with poor outcome, recent randomized controlled trials (RCTs) have shown that early full nutritional support does not benefit critically ill patients and may induce dose-dependent harm. Some researchers have suggested that the absence of benefit in RCTs may be attributed to overrepresentation of patients deemed at low nutritional risk, or to a too low amino acid versus non-protein energy dose in the nutritional formula. However, these hypotheses have not been confirmed by strong evidence. RCTs have not revealed any subgroup benefiting from early full nutritional support, nor benefit from increased amino acid doses or from indirect calorimetry-based energy dosing targeted at 100% of energy expenditure. Mechanistic studies attributed the absence of benefit of early feeding to anabolic resistance and futile catabolism of extra provided amino acids, and to feeding-induced suppression of recovery-enhancing pathways such as autophagy and ketogenesis, which opened perspectives for fasting-mimicking diets and ketone supplementation. Yet, the presence or absence of an anabolic response to feeding cannot be predicted or monitored and likely differs over time and among patients. In the absence of such monitor, the value of indirect calorimetry seems obscure, especially in the acute phase of illness. Until now, large feeding RCTs have focused on interventions that were initiated in the first week of critical illness. There are no large RCTs that investigated the impact of different feeding strategies initiated after the acute phase and continued after discharge from the intensive care unit in patients recovering from critical illness.

## Background

In critically ill patients, severe physical stress induces a catabolic response, leading to muscle wasting and weakness [1]. The longer the stay in the intensive care unit (ICU), the higher the risk of weakness, and the poorer the outcome [1, 2]. Indeed, severe weakness may preclude weaning from mechanical ventilation and may cause life-threatening complications by difficulties to cough up secretions and swallowing dysfunction, among others [1]. Also in patients surviving critical illness, persistent weakness is considered part of the post-intensive care syndrome [3–5]. Apart from weakness, also increased bone resorption may occur, with increased fracture risk after intensive care [5–7].

To counteract catabolism, nutrition has been advocated, since prolonged underfeeding could contribute to catabolism [8]. Moreover, some patients already have sarcopenia and prolonged low nutrition intake before ICU admission. Numerous observational studies have associated increased nutrition intake with improved outcome of critically ill patients [9, 10]. Yet, a causal relationship cannot be derived from such associations, since feeding tolerance closely associates with illness severity, with in general a better feeding tolerance in patients who are less ill. Hence, in observational studies, there is an inherent risk of residual confounding. Until a decade ago, in view of the absence of large randomized controlled trials (RCTs), European experts advocated to avoid any caloric or protein deficit in critically ill patients, and to start early artificial feeding, especially in patients considered to be at high nutritional risk [8]. Since then, however, several large RCTs have shown that early full feeding did not benefit adult and pediatric critically ill patients, and some even showed harm [11–15]. These at first sight counterintuitive results indicate that critical illness-associated catabolism is much more complex than merely a consequence of underfeeding, and that anorexia and temporary starvation may to some extent be an adaptive component of the stress response to severe illness. We here summarize the RCT evidence and review potential mechanisms explaining the negative results of recent feeding RCTs, which will hopefully guide future research and which may ultimately lead to individualization of feeding.

### The impact of early full feeding in critical illness: evidence from recent feeding RCTs

As shown in a recent meta-analysis, no large-scale RCT in critically ill patients found benefit by early full feeding, as compared to more restrictive feeding regimens [16]. Two RCTs—one in adults and one in children—even found significant harm by early supplementation of insufficient or contraindicated enteral nutrition with parenteral nutrition. Indeed, in both the adult EPaNIC (*N* = 4640) and pediatric PEPaNIC RCTs (*N* = 1440), providing early supplemental parenteral nutrition prolonged ICU dependency, with increased dependency on vital organ support and incidence of new infections as compared to withholding supplemental parenteral nutrition until one week after ICU admission [11, 12]. In adults, early supplemental parenteral nutrition further increased the incidence of ICU-acquired weakness, and hampered recovery hereof [17]. In theory, harm by early supplemental parenteral nutrition could be explained by an increased nutritional dose, or by an inferior feeding route. However, 2 large RCTs in adults—the CALORIES (*N* = 2400) and Nutrirea-2 (*N* = 2410) RCT—showed no harm by parenteral nutrition when provided at isocaloric doses as enteral nutrition [18, 19], suggesting that harm by early supplemental parenteral nutrition in the EPaNIC and PEPaNIC RCTs is explained by the higher nutritional dose, rather than by the intravenous route. Moreover, the large-scale EDEN (*N* = 1000), PermiT (*N* = 894) and TARGET (*N* = 3957) RCTs, which compared early full enteral nutrition with lower-dose enteral nutrition for 6, respectively 14 or 28 days in ICU in critically ill adults, did not find benefit with higher nutritional doses [13–15].Two of these RCTs found more gastrointestinal intolerance with early full enteral nutrition [13, 15]. Also in long-term follow-up, providing early enhanced nutrition was not beneficial with regard to functional outcome [20–24]. Of note, the EPaNIC and PEPaNIC RCTs showing harm by early enhanced feeding had the highest relative difference in caloric intake between the 2 study groups, which enhances the statistical power to detect a treatment effect [25].

Based on this recent high level evidence, the most recent European feeding guidelines for adult critically ill patients shifted from promoting early full feeding to less aggressive artificial feeding in the first week of critical illness [26]. However, it is important to note that the shift toward providing less feeding in the acute phase should not increase the risk of refeeding syndrome, which is caused by a deficiency in micronutrients and electrolytes, including vitamin B1, potassium and phosphate [27]. Indeed, when artificial feeding is restarted after a prolonged period of starvation, the metabolic need and intracellular transport of several micronutrients and electrolytes increases, which may unmask preexisting deficiencies and lead to life-threatening symptoms [27]. A biochemical hallmark of this condition is refeeding hypophosphatemia, which has been defined as a drop in phosphate levels below 0.65 mmol/l within 72 h after institution of artificial feeding [28, 29], explained by intracellular uptake and incorporation in energy-rich phosphate bonds. Once refeeding hypophosphatemia occurs early in critical illness, temporarily limiting nutrition intake while correcting existing vitamin and electrolyte deficiencies is likely beneficial, as shown in the Refeeding RCT (*N* = 339) [28]. To prevent refeeding syndrome, it seems prudent to ensure sufficient micronutrient intake in all patients, which may, especially in the acute phase of illness, require parenteral administration of micronutrients and electrolytes [11, 26, 30].

### Critiques on recent feeding RCTs

The neutral or negative effect of early enhanced feeding in recent RCTs has been suggested to be explained by over-representation of patients presumed to carry low risk of malnutrition, by administration of too low amino acid doses, and by the use of calculated energy targets [31–33]. However, these critiques have not been supported by high level evidence and several lines of evidence have contradicted them, as outlined below. The Nutrirea-3 RCT, which randomized 3044 adult patients with shock requiring mechanical ventilation and vasopressor support to early full feeding versus one week of calorie-protein restriction irrespective of the feeding route, recently finalized recruitment and will provide more insight (NCT03573739) [34].

#### Inclusion of patients presumed to be at low risk of malnutrition

Researchers have suggested that the absence of benefit in recent RCTs may be explained by including too many patients considered to be at low risk of malnutrition, whereby any potential benefit in perceived high-risk patients may have been obscured by no impact or even harm in hypothesized low-risk patients [32]. However, this hypothesis is not confirmed by subgroup analyses of RCTs. Indeed, in large RCTs, there was no subgroup of patients identifiable who benefited from early enhanced feeding, as defined by age, the nutritional risk screening (NRS) score, the modified Nutrition Risk in Critically Ill (NUTRIC) score, or body mass index (BMI) upon admission [11–13, 15, 35]. In a secondary analysis of the PermiT RCT, studied biomarkers did not discriminate adult patients who would benefit from early full enteral nutrition as compared to lower-dose enteral nutrition [35]. If anything, there was a signal in the opposite direction. Indeed, patients with low prealbumin levels, who would be considered at highest nutritional risk, had an increased risk of mortality associated with early full enteral nutrition [35]. Also in the large EPaNIC subgroup of patients for whom enteral nutrition was contraindicated (*N* = 517), early total parenteral nutrition was harmful as compared to virtual starvation for one week in ICU [11].

#### Low amino acid doses

It has been suggested that several feeding RCTs did not show benefit because of imbalanced feeding solutions, whereby the doses of amino acids would have been too low [31]. However, the largest RCT on amino acid supplements in adult critically ill patients, the Nephroprotective RCT (*N* = 474), did not find benefit from early amino acid supplements provided at doses of approximately 1.75 g/kg per day throughout ICU stay, while significantly increasing ureagenesis [36]. Also, in other RCTs in both adults and children, early full feeding significantly increased ureagenesis [37–39]. In a secondary analysis of the EPaNIC RCT, it was estimated that approximately two third of the extra amino acids provided through early parenteral nutrition were net wasted in ureagenesis, even with amino acid doses that are considered relatively low (approximately 0.8 g/kg per day) [37]. Concomitantly, both microscopic and macroscopic muscle loss were not prevented by providing early full feeding [17, 40]. In contrast, early supplementation of insufficient enteral nutrition by parenteral nutrition increased muscular fat content, aggravated muscle weakness, and hampered recovery from weakness [17]. In secondary analyses of both EPaNIC and PEPaNIC RCTs, harm by early parenteral nutrition was statistically explained by the amino acid doses, and not by the glucose or lipid doses [38, 41]. Evidently, these findings are observational and require confirmation in RCTs. Of note, in the absence of solid evidence supporting a clear protein target, the most recent European ESPEN guidelines for adult critically ill patients do not make a strong recommendation [26]. Instead, there is a grade 0 recommendation suggesting that 1.3 g/kg protein can be delivered progressively [26]. The EFFORT RCT (*N* = 4000; study completed December 3, 2021, with 1329 patients included according to clinicaltrials.gov) will fill this evidence gap, as it investigates whether or not a higher dose of proteins improves outcome of adult critically ill patients [42].

#### Calculated energy targets

The absence of benefit of early full feeding has also been attributed to the absence of indirect calorimetry to guide the energy target [43]. In acute illness, indirect calorimetry is the gold standard to measure energy expenditure, which is derived from measurement of VO2 and VCO2, and the obtained value has been proposed as energy target after the first days in ICU [44]. In most recent large feeding RCTs, indirect calorimetry was not routinely used, reflecting daily practice in most centers [11–15]. Instead, the energy target was determined by predictive equations that only provide an estimation of energy expenditure that may considerably deviate from the measured energy expenditure [11–15, 44]. However, there is no solid evidence that the feeding target should equal energy expenditure at all times, since the largest RCTs comparing indirect calorimetry-based feeding versus predictive equation-based feeding in adult critically ill patients did not show clear benefit [45, 46]. The EAT-ICU RCT (*N* = 199) even found harm, with an increased ICU stay in patients randomized to the intervention group in which early full feeding was guided by indirect calorimetry and by nitrogen balances as compared with the control group in which early enteral nutrition was delivered up to a fixed energy target [39]. Interestingly, the EAT-ICU intervention resulted in higher protein and energy intake in the first week than the control group, further supporting harm by a higher nutritional dose early during critical illness [39].

Despite the absence of benefit from using indirect calorimetry to target 100% of energy expenditure by feeding in large RCTs, proponents of its use in the first week of critical illness have referred to the results of a recent meta-analysis that suggested potential mortality benefit by indirect calorimetry-based feeding initiated in the first week in critically ill adults as compared with calculated energy target-based feeding [47, 48]. The potential mechanisms of mortality benefit remain unclear, however, since morbidity outcomes did not differ [47]. Although this meta-analysis may seem encouraging, the results should be interpreted with great caution, for several reasons. First, none of the included studies had a low risk of bias, and the mortality difference was barely significant [47]. It is highly likely that the statistical difference would be lost if a small number of patients (close to 1)—the fragility index of the study—would have had a different outcome. Moreover, there are concerns with regard to the reported mortality data in the largest RCT, the TICACOS-International RCT (*N* = 417) [46, 49]. Indeed, the reported mortality at consecutive time points decreased over time in this RCT, which is obviously impossible, and reported numbers in abstract and full text do not match [46], as reported in a letter to the editor [49]. Moreover, according to the reported numbers in TICACOS-International, there was only a statistically insignificant, but numerical difference in mortality at 90 days, which was the mortality rate used in the meta-analysis [46, 47]. In contrast, reported ICU mortality and mortality at 6 months, not used in the meta-analysis, were virtually identical [46, 47]. In view of these important concerns and unresolved issues, the level of evidence put forward by the meta-analysis remains low. Moreover, the TICACOS-International RCT may indirectly question the feasibility of widespread implementation of indirect calorimetry, since the authors, who are experts in the field, only included 417 patients over 6 years in 7 centers, whereby slow recruitment led to premature stopping of the RCT [46].

**Fig. 1:**
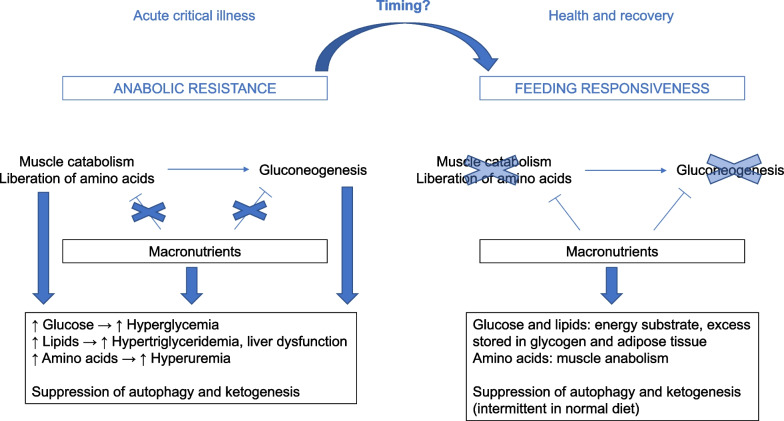
Selected mechanisms explaining the lack of benefit by early full feeding in critical illness. Evoked by the stress response to severe illness, anabolic resistance occurs, whereby muscle catabolism and hepatic gluconeogenesis cannot be counteracted by providing macronutrients, unlike in normal health. Providing extra macronutrients in such condition increases the risk of overfeeding, manifested as hyperglycemia, hypertriglyceridemia, liver dysfunction and hyperuremia by catabolism of extra provided amino acids. In addition, continuous artificial nutrition continuously suppresses autophagy and ketogenesis as potentially important repair pathways. The time when anabolic resistance ceases and the condition reverses into metabolic feeding responsiveness cannot be predicted or monitored at the bedside. Theoretically, feeding responsiveness may undergo dynamic changes over time, and the timing of such changes likely differs between patients

Apart from the absence of benefit from full feeding guided by indirect calorimetry in the largest RCTs, there are also pathophysiological concerns with regard to its early use to guide nutritional energy dosing. Indeed, if the ideal energy target would equal energy expenditure at all times, one intrinsically assumes that all endogenous energy production can be suppressed by providing calories by feeding, which is not the case (Fig. 1). Indeed, acute critical illness is characterized by feeding-resistant catabolism and severe insulin resistance, especially in the liver, whereby endogenous glucose production cannot be suppressed by providing nutrients and insulin [50]. Hence, providing extra calories on top of not suppressible gluconeogenesis may aggravate hyperglycemia and hypertriglyceridemia, and may only pose an additional burden on the liver [51]. Unfortunately, there is no monitor of endogenous glucose production available at the bedside, so the duration and extent of unsuppressible endogenous substrate production of individual patients remain unclear. There are no RCTs that investigated the impact of reducing feeding intake to a fixed percentage of the measured energy expenditure, to compensate for endogenous glucose production [52]. Also, no large RCTs have investigated the impact of indirect calorimetry-based feeding that is initiated in prolonged critically ill patients and continued after ICU discharge. Of note, the current ESPEN guidelines and recent experts’ opinion do not recommend to match the energy expenditure measured by indirect calorimetry with the feeding target at all times in adult critically ill patients [26, 53].

### Mechanisms explaining lack of benefit of early full feeding

#### Suppression of fasting-induced recovery pathways

The lack of benefit from early full nutrition in RCTs may be explained by a continuous suppression of the fasting response. Although fasting has traditionally been considered a negative process in critical illness [8], a normal diet involves alteration of feeding periods with fasting intervals, and fasting has been attributed health-promoting effects. Indeed, diets that induce a prolonged fasting response such as fasting-mimicking diets or caloric restriction diets protected against age-related disease and improved longevity in animal models, and ameliorated risk factors of age-related disease in humans [54, 55]. This suggests that fasting-activated pathways are important to maintain normal cellular integrity and function. In large feeding RCTs in critically ill patients, however, artificial feeding has always been provided in a continuous manner [11–15], hereby continuously suppressing any fasting response.

Part of the beneficial effects of fasting in normal health are mediated by activation of macroautophagy [54]. Macroautophagy, hereafter referred to as autophagy, is a cellular process whereby cytoplasmic content is digested in the lysosome after its delivery to the lysosome in an intermediate vesicle that is called an autophagosome [56]. Autophagy is activated by fasting and by a variety of stress signals [56]. Particularly deprivation of amino acids is a strong stimulus of autophagy [56]. Autophagy is the only process able to remove macromolecular damage, including damaged organelles, potentially toxic protein aggregates and intracellular microorganisms and as such, it is a crucial process that is necessary to maintain homeostasis [57]. Aging is accompanied by a gradual decline in autophagic activity, and activation of autophagy has been shown to protect against age-related disease and to improve life span in animals [57, 58]. Increasing evidence also implicates autophagy as crucial repair process to recover from critical illness [59–61]. Moreover, mechanistic studies have implicated autophagy suppression as potential mechanism explaining harm by early full nutrition in critical illness [62]. In a critically ill animal model, early parenteral nutrition, especially with higher amino acid doses, increased liver damage and signs of muscle degeneration as compared to relative fasting, while it suppressed autophagy [63]. In this model, administration of the autophagy activator rapamycin protected against kidney injury in fed critically ill animals [64]. Likewise, in critically ill patients, early parenteral nutrition suppressed autophagy in muscle, which associated with more weakness [17]. Altogether, this evidence puts forward autophagy as potential therapeutic target in critical illness. However, pharmacological autophagy activation is complicated, since there are no specific pharmacological autophagy inducers available [65], and excessive autophagy stimulation may also be detrimental [66].

A second process that may explain the negative impact of early full feeding in critically ill patients is suppression of ketogenesis. Apart from being an alternative energy substrate during fasting, ketones serve signaling roles, may stimulate autophagy and enhance muscle regeneration [67, 68]. In a mouse model of sepsis-induced critical illness, administration of ketones improved muscle force, which appeared not related to its use as energy substrate, but by activating muscle regeneration pathways [68]. A recent study showed that ketones increase resilience of muscle stem cells to cellular stress via signaling effects [69]. Secondary analyses of the EPaNIC and PEPaNIC RCTs showed that withholding early parenteral nutrition activated ketogenesis, most robustly in critically ill children, in whom it statistically mediated part of the outcome benefit of the intervention [70, 71].

#### Anabolic resistance

One of the main aims of providing nutrients to critically ill patients is to inhibit or limit critical illness-associated catabolism, which would attenuate muscle wasting and weakness, and improve long-term functional outcome. However, recent nutritional RCTs have shown that early full feeding is unable to counteract catabolism. Indeed, both muscle wasting and weakness were not prevented, and long-term functional outcome was not improved [17, 20–22, 40]. Instead, providing higher doses of amino acids in the acute phase significantly increased ureagenesis in several RCTs [36–39]. Currently, there are no bedside monitors or biomarkers that predict or document feeding responsiveness. The failure of artificial feeding to suppress catabolism may to some extent be explained by the so-called muscle-full effect [72, 73]. Indeed, in healthy adults, muscle protein synthesis only rises temporarily in response to continuous amino acid infusion [72]. However, whether bolus feeding or intermittent feeding would indeed lead to more anabolism in critically ill patients remains to be studied. A relatively small RCT in critically ill adults (*N* = 121) found lower rise in the urea over creatinine ratio as marker of catabolism by intermittent feeding as compared to continuous feeding, while there was no impact on ultrasound-assessed muscle wasting [74, 75]. Moreover, there was already a baseline difference in urea over creatinine ratio, precluding a strong conclusion [75]. Regardless of the mode of delivering nutrition, the degree of anabolic resistance likely varies over time and among patients, since critical illness-associated catabolism has been related to the stress response and the accompanying inflammatory and endocrine alterations (Fig. 1) [1]. Apart from the muscle-full effect, another potential mechanism contributing to anabolic resistance is the relative immobilization of the patient. Outside critical illness, protein supplementation is most effective in achieving anabolism when combined with exercise [76]. There are no data regarding early mobilization in large feeding RCTs in critical illness, and large RCTs investigating the interaction between early mobilization and feeding in critically ill patients are lacking [77]. However, as for early feeding, early enhanced, active mobilization is not beneficial for critically ill patients and increases the risk of adverse events [78].

### Perspectives for future research

These mechanistic insights provide a base for novel feeding regimens, to be developed and to be tested ultimately in RCTs powered for clinical endpoints. Although fasting may activate beneficial cellular pathways that are also essential in normal health, prolonged starvation will likely come at a price. Novel feeding strategies that may exploit these fasting-associated benefits while avoiding prolonged starvation include intermittent feeding, ketogenic diets and ketone supplementation.

Intermittent feeding diets, which alternate feeding with fasting intervals, would theoretically allow to provide feeding while intermittently activating the fasting response and its associated benefits [79]. In animal models of aging, so-called fasting-mimicking diets could replicate the benefits observed with caloric restriction [80]. Apart from activating fasting responses, intermittent feeding strategies could theoretically be beneficial through preventing the muscle-full effect and better preservation of circadian rhythm [79]. However, it remains unclear how long critically ill patients should fast before a metabolic fasting response that includes autophagy stimulation develops [81]. In a pilot crossover RCT, 12 h fasting activated ketogenesis and other components of the fasting response, while it had no impact on autophagy assessed in peripheral blood cells [82]. Yet, it remains unclear whether 12 h fasting was able to activate autophagy in vital tissues, or whether 12 h fasting was merely insufficient to initiate autophagy stimulation at all [82]. RCTs investigating the impact of intermittent versus continuous feeding strategies in critical illness did not show consistent benefit of intermittent feeding [74, 83]. Yet, RCTs were relatively small and likely underpowered to detect or exclude a meaningful clinical benefit, and the fasting interval was relatively short (in general 4–6 h), which may have been too short to induce a fasting response and its associated benefits [79]. Nevertheless, intermittent feeding may also be challenging, since the daily nutritional intake has to be given over a shorter time, which may increase the risk of complications due to enteral feeding intolerance and large glucose variability, among others [84]. Hence, efficacy and safety remain to be studied. Apart from intermittent feeding strategies, ketogenic diets or ketone supplementation could be beneficial [85]. Although ketogenic diets have been used in selected patients including patients with refractory status epilepticus, the efficacy and safety of ketogenic diets or ketone supplements for general critically ill patients remain to be studied [85].

Apart from the ideal feeding regimen or the ideal feeding mode, there is a need for validated markers of feeding tolerance and responsiveness [86]. Indeed, although enteral nutrition is usually favored over parenteral nutrition, patients on enteral nutrition may suffer feeding intolerance and, in severe cases, non-occlusive mesenteric ischemia, especially when delivered at higher doses in patients with shock [19, 86]. Currently, there are no validated biomarkers or bedside monitoring devices that can predict enteral feeding tolerance, which could help avoid complications of too early enteral feeding, such as aspiration pneumonia [87, 88]. At current, gastric residual volumes are still widely used and recommended by guidelines [89], although a RCT (*N* = 449) did not show benefit of measuring gastric residual volumes in adult patients receiving mechanical ventilation [90]. Also, metabolic responsiveness to feeding cannot be predicted or monitored at the bedside, which requires further investigation (Fig. 1) [87]. In the past, experts have recommended to use nutritional risk scores to inform which patients would benefit most from early enhanced nutrition [91, 92]. However, RCT data have shown that no biomarker was able to discern subpopulations of patients benefiting from early full nutrition [35]. Future research in metabolomics could help to identify which patients may benefit from enhanced or more restricted feeding and at what time [93, 94]. Currently used signs of energy or protein overload are nonspecific and frequently occur outside the context of overfeeding, including hyperglycemia, hypertriglyceridemia, elevated liver enzymes, hyperbilirubinemia, hyperuremia and hyperammonemia [95]. A potential sign that may assist in determining readiness for feeding may be the degree of insulin resistance, as can be derived from the amount of insulin required to maintain blood glucose at a predefined level [95, 96]. One important biomarker is, however, phosphate, to detect and early treat refeeding syndrome [95].

The target population and outcomes studied in RCTs on ICU nutrition also need to be considered. As responsiveness to feeding likely changes over time, there is a need for RCTs that investigate the impact of optimized nutrition started after the acute phase and continued throughout the recovery phase [97], since anabolic resistance is expected to cease at a particular time. In this regard, a large RCT in hospitalized non-critically ill adults at risk of malnutrition (*N* = 2088) showed that intensified nutritional support, achieved predominantly through increased oral intake, improved short-term mortality [98]. Nevertheless, the mortality difference was only transient [99], there was no impact on functional outcome after 6 months [99], and less than 2% of patients in the intervention group received enteral or parenteral nutrition [98]. Hence, it is not clear to what extent these findings can be extrapolated to patients who are still in need of artificial nutrition while recovering from critical illness. Theoretically, indirect calorimetry could be a useful adjunct in patients who are responsive to feeding, to prevent over- and underfeeding. Yet, feeding responsiveness cannot be monitored at the bedside at this time.

In future nutritional RCTs, the use of uniform endpoints would facilitate comparisons and meta-analyses, although there is only limited agreement on essential outcomes [100]. There has been considerable variability in the primary and secondary outcomes of RCTs [101, 102]. For large efficacy RCTs, the primary endpoint should be a patient-centered outcome that is likely affected by feeding [101]. The anticipated effect size should be realistic with regard to the nature and the duration of the intervention, avoiding an underpowered study. Evidently, potential confounders should be taken into account, including competing risks [101, 103].

## Conclusion

Recent RCTs have not confirmed the hypothesized benefit of early full feeding, and several RCTs even showed harm of early parenteral nutrition supplementing insufficient enteral nutrition. Harm by early parenteral nutrition appeared explained by a higher nutritional dose in the acute phase, and not by the parenteral route per se, since a short period of parenteral nutrition did not cause harm as compared to an isocaloric dose of enteral nutrition. There are no large RCTs favoring indirect calorimetry-guided full feeding as compared to calculation-based feeding. The absence of benefit of early full feeding has been attributed to suppression of autophagy and ketogenesis, and to feeding-resistant muscle catabolism. Hence, intermittent feeding, ketone supplementation and ketogenic diets emerge as potential novel feeding strategies that may allow continuation of nutrition while avoiding prolonged suppression of beneficial fasting responses, which needs further study. Despite many large-scale RCTs in the last decade, it remains unclear how to optimally administer feeding, since the ideal timing and dose remain unclear. Since recent feeding practices have shifted toward lower-dose artificial nutrition and avoiding early PN, sufficient micronutrient intake should be ensured to prevent deficiencies. To allow individualization of feeding, novel biomarkers, predictive models or monitoring devices that predict and indicate the response to feeding are needed, since the presence or absence of feeding resistance and unsuppressible gluconeogenesis is likely dynamic and time-dependent, depending on the stress response to severe illness and the recovery hereof. Until that time, it will remain unclear for whom, when and how to optimally use indirect calorimetry. Evidently, any feeding strategy, even in case of a solid pathophysiological rationale, requires confirmation of efficacy and safety in a large-scale RCT powered for clinical endpoints before it can be strongly recommended in clinical practice.

## Data Availability

Data sharing is not applicable to this article as no datasets were generated or analyzed during the current study.

## Abbreviations

BMI: Body mass index
ESPEN: European Society for Clinical Nutrition and Metabolism
ICU: Intensive care unit
NRS: Nutritional risk screening
NUTRIC: Nutrition risk in critically ill
RCT: Randomized controlled trial
